# Α very rare case of De Garengeot’s hernia: Acute appendicitis within a femoral hernia

**DOI:** 10.1002/ccr3.1914

**Published:** 2018-11-16

**Authors:** Nikolaos G. Filippou, Domna Fanidou, Georgios Alvanos, Georgios Ouilson Papadopoulos, Dimitrios Filippou, Panagiotis Skandalakis

**Affiliations:** ^1^ 2^nd^ Surgical Department Athens General Hospital “G. Gennimatas’’ Athens Greece; ^2^ Department of Anatomy and Surgical Anatomy, Medical School National and Kapodestrian University of Athens Athens Greece; ^3^ Department of Plastic Surgery Athens General Hospital “Evangelismos” Athens Greece

**Keywords:** appendicitis, De Garengeot, incarcerated femoral hernia, s hernia

## Abstract

De Garengeot's hernia is a rare clinical entity. Appropriate and acute diagnosis in emergency basis is challenging (usually misdiagnosed as incarcerated femoral hernia), and the surgical management varies from case to case. This report emphasizes the importance of including De Garengeot's hernia in the differential diagnosis of incarcerated groin hernias and the need to establish a well‐defined strategy regarding surgical management options.

## 1 INTRODUCTION

Inflamed appendix within a femoral hernia is an extremely rare clinical entity representing less than 1% of all femoral hernias. Prompt diagnosis on emergent basis is challenging (usually misdiagnosed as incarcerated femoral hernia), and the surgical management varies from case to case.

We present the case of a 77‐year‐old woman who presented in the emergency department complaining of enlarging tender mass in the right groin. A CT of the abdomen‐pelvis showed the top of the appendix in the femoral sac with signs of inflammation. The patient underwent appendectomy and hernioplasty through the femoral sac without any complications. This report emphasizes the importance of including De Garengeot's hernia in the differential diagnosis of incarcerated groin hernias and the need to establish a well‐defined strategy regarding surgical management options.

The femoral canal is anatomically located in the groin, medially to the vascular compartment containing the femoral artery and vein. It is a funnel‐shaped canal, approximately 1‐2 cm in length and 10‐20 mm in diameter, defined laterally by the sheath of the femoral vein, medially the aponeurosis of the transversus abdominis, anteriorly the inguinal ligament and posteriorly the pectineal ligament of Cooper.^1^ The protrusion of abdominal content through the femoral canal is called femoral hernia. Femoral hernias account for 3% of hernias and are more common in females.^2^, ^3^ The high prevalence among women is attributed to collagen defects, increased intraabdominal pressure, and body changes during pregnancy.^2^ In less than 3% of the femoral hernias, the appendix protrudes through the canal. This rare clinical entity is called De Garengeot's hernia, named after the French surgeon who first described it in 1731.^4^ The incidence of the appendix being inflamed is even less common. In this report, we present a case of De Garengeot's hernia complicated by appendicitis, managed with emergent appendectomy, and hernioplasty through herniotomy.

## 2 CASE REPORT

**Figure 1:**
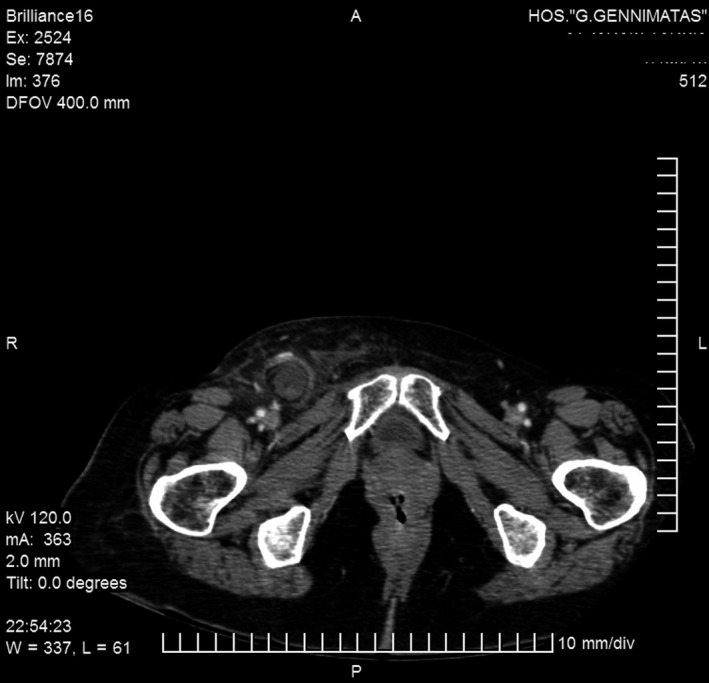
Preoperative CT scan of the abdomen‐pelvis showing a right femoral hernia containing the swollen appendix and fluid collection in the femoral canal

A 77‐year‐old woman presented in the emergency department complaining of enlarging painful mass in the right groin for the past 2 days. She denied any nausea, emesis, diarrhea, or constipation. The patient was afebrile upon presentation, and the rest of her vital signs were normal. Physical examination revealed a bulging tender mass in the right groin consistent with femoral hernia, which was non‐reducible. Laboratories were remarkable for elevated WBC count (13 500/μL) and CRP (34 mg/L). A CT of the abdomen‐pelvis showed the top of the inflamed appendix within the femoral sac and a small fluid collection in the femoral canal area (Figure 1). A diagnosis of De Garengeot's hernia was made, and the patient was transferred to the operating room. After administration of preoperative antibiotics (cefuroxime 1500 mg, metronidazole 500 mg), a small incision was made (approximately 4 cm) beneath the inguinal ligament under general anesthesia (Lockwood's infrainguinal approach).^5^ The femoral sac was carefully dissected to the level of the femoral orifice. The sac was opened, and the inflamed appendix was visualized. The cecum was mobile and easily identified and appendectomy was performed. The hernia sac was reduced and the boundaries of the defect carefully defined. No signs of abscess or perforation were noted, and the patient's wall abdominal condition made a suture repair technique inefficient. Thus, a polypropylene mesh plug was fixed into the defect (with interrupted nonabsorbable prolene sutures) to the inguinal ligament, the Cooper ligament, and the aponeurosis of the transversus abdominis. Subcutaneous tissue and skin were closed after good hemostasis. No postoperative complications were noted. The patient was discharged 7 days after the operation, and complete wound closure was noted during follow up. The pathologic examination of the surgical specimen confirmed the diagnosis of mucosal inflammation of the vermiform appendix.

## 3 DISCUSSION

De Garengeot's hernia is a rare clinical entity that should be suspected especially in elderly patients with clinical signs of nonreducible, tender femoral hernia. The differential diagnosis from incarcerated femoral hernia is difficult, can be facilitated by imaging and is sometimes achieved intraoperatively. Imaging findings on abdominal CT include the presence of the appendix within the femoral sac and signs of inflammation in the femoral canal. Complications of untreated De Garengeot's hernia include necrosis of the hernia contents with perforation and sepsis or abscess formation,^5^, ^6^ bowel obstruction,^7^ and necrotizing fasciitis.^2^ These complications highlight the perils of misdiagnosis leading to inappropriate or delayed management. In regard to the surgical management, there is currently no formal strategy established. Several approaches have been reported with minimal postoperative complications and comparable results. Laparoscopic appendectomy and hernioplasty via anterior approach is recommended in some cases in order to prevent surgical site infection and mesh contamination.^5^, ^8^ Simple suture hernioplasty instead of mesh repair is suggested by a number of authors for the same reason.^5^, ^9^ In our case, we performed the appendectomy and the mesh hernioplasty through the same incision without any signs of surgical site contamination. A mesh can be used, if there is no abscess or appendiceal perforation, without increasing the risk of infection or hernia recurrence. The presence of field contamination and the status of the abdominal wall are crucial factors while establishing the treatment plan. The use of ePTFE patch instead of a polypropylene mesh is suggested because such materials are less prone to inflammation and can be removed more easily.^10^ The infrainguinal approach was used in our case with no need for further exposure, because the cecum and appendix were easily visualized. If the base of the appendix is not safely accessed, peritoneal entry may be gained by creating a plane superficial to the external oblique, division of linea semilunaris, medial retraction of rectus muscle, and incision of transversalis fascia and peritoneum.^5^

## 4 CONCLUSION

De Garengeot's hernia is a rare type of femoral hernia that contains the appendix and can be complicated by appendicitis. High clinical suspicion is required for early diagnosis and prompt surgical management in order to avoid further complications. The surgeon should feel free to choose the surgical approach appropriate for each case, depending on the preoperative and intraoperative findings.

## INFORMED CONSENT

An informed consent form from the patient in order to present the case for scientific purposes has been obtained.

## CONFLICT OF INTEREST

None declared.

## AUTHOR CONTRIBUTION

NF: is the corresponding author; a member of the surgical team; involved in the study, design, and analysis of data; drafted and critically revised the manuscript. DF: is a member of the surgical team: involved in the study, design, and analysis of data; drafted and critically revised the manuscript. GA: is a member of the surgical team; involved in critical revision of the manuscript. GOP: involved in the study, design, analysis and interpretation of data; drafted the manuscript. DF: involved in the design and analysis of data; drafted and critically revised manuscript. PS: supervised the drafting and critical revision of the manuscript.
